# Clinical characteristics and prognosis of temporary miller fisher syndrome following COVID-19 vaccination: a systematic review of case studies

**DOI:** 10.1186/s12883-023-03375-4

**Published:** 2023-09-21

**Authors:** Dorsa Alijanzadeh, Afsaneh Soltani, Fatemeh Afra, Fardis Salmanpour, Amir Hossein Loghman, Noosha Samieefar, Nima Rezaei

**Affiliations:** 1 Student Research Committee, School of Medicine, Shahid Beheshti University of Medical Sciences, Tehran, Iran; 2 Network of Interdisciplinarity in Neonates and Infants (NINI), Universal Scientific Education and Research Network (USERN), Tehran, Iran; 3 USERN Office, Shahid Beheshti University of Medical Sciences, Tehran, Iran; 4 Clinical Pharmacy Department, Faculty of Pharmacy, Tehran University of Medical Sciences (TUMS), Tehran, Iran; 5 Student Research Committee, School of Medicine, Ahvaz Jundishapur University of Medical Sciences, Ahvaz, Iran; 6 School of Medicine, Kashan University of Medical Sciences, Kashan, Iran; 7 Research Center for Immunodeficiencies, Children’s Medical Center Hospital, Tehran University of Medical Sciences, Tehran, Iran; 8 Department of Immunology, School of Medicine, Tehran University of Medical Sciences, Tehran, Iran

**Keywords:** COVID-19 vaccine, Miller-Fisher syndrome, MFS, Ophthalmoplegia, Ataxia, Areflexia, Guillain– Barré syndrome, SARS-CoV-2

## Abstract

**Background:**

Miller Fisher syndrome (MFS) is a subtype of Guillain-Barré syndrome (GBS) which is characterized by the three components of ophthalmoplegia, ataxia, and areflexia. Some studies reported MFS as an adverse effect of the COVID-19 vaccination. We aimed to have a detailed evaluation on demographic, clinical, and para-clinical characteristics of subjects with MFS after receiving COVID-19 vaccines.

**Materials and methods:**

A thorough search strategy was designed, and PubMed, Web of Science, and Embase were searched to find relevant articles. Each screening step was done by twice, and in case of disagreement, another author was consulted. Data on different characteristics of the patients and types of the vaccines were extracted. The risk of bias of the studies was assessed using Joanna Briggs Institute (JBI) tools.

**Results:**

In this study, 15 patients were identified from 15 case studies. The median age of the patients was 64, ranging from 24 to 84 years. Ten patients (66.6%) were men and Pfizer made up 46.7% of the injected vaccines. The median time from vaccination to symptoms onset was 14 days and varied from 7 to 35 days. Furthermore,14 patients had ocular signs, and 78.3% (11/14) of ocular manifestations were bilateral. Among neurological conditions, other than MFS triad, facial weakness or facial nerve palsy was the most frequently reported side effect that was in seven (46.7%) subjects. Intravenous immunoglobulin (IVIg) was the most frequently used treatment (13/15, 86.7%). Six patients received 0.4 g/kg and the four had 2 g/kg. Patients stayed at the hospital from five to 51 days. No fatal outcomes were reported. Finally, 40.0% (4/15) of patients completely recovered, and the rest experienced improvement.

**Conclusion:**

MFS after COVID-19 immunization has favorable outcomes and good prognosis. However, long interval from disease presentation to treatment in some studies indicates that more attention should be paid to MFS as the adverse effect of the vaccination. Due to the challenging diagnosis, MFS must be considered in list of the differential diagnosis in patients with a history of recent COVID-19 vaccination and any of the ocular complaints, ataxia, or loss of reflexes, specially for male patients in their 60s and 70s.

**Supplementary Information:**

The online version contains supplementary material available at 10.1186/s12883-023-03375-4.

## Introduction

Miller-Fisher syndrome (MFS) is a variant of Guillain–Barré syndrome (GBS), which is presented as the clinical triad of ataxia, areflexia, and ophthalmoplegia [1]. About half of the patients might experience symptoms related to the cranial nerves involvement other than the oculomotor nerves [2]. Previous *Campylobacter jejuni* or *Hemophilus influenza* infections are reported to be associated with the MFS onset [3, 4]. MFS is previously reported to be a side effect of vaccine administration, which may occur from 5 to 21 days after immunization with pneumovax [5], influenza [6], and Diphtheria—Pertussis—Tetanus (DPT) vaccines [7]. The molecular mimicry of infective agents bearing the GQ1b epitope is a described pathogenic mechanism of the MFS [3]. Notably, foreign antigens are suggested to stimulate an abnormal immune response, which targets the gangliosides found in peripheral nerves [8].

By introducing COVID-19 vaccines in early 2021, mortality rates of the infection were significantly reduced [9]. However, some incidental neurological complications in subjects after receiving COVID-19 vaccines have been reported [10, 11]. Reactivation of herpes zoster, GBS, and acute disseminated encephalomyelitis are among the instances [12]. Importantly, the reporting rate of GBS after COVID-19 vaccination is shown to be significantly higher than the rates after other vaccinations [13].

Though several reports are available about patients experiencing MFS after COVID-19 immunization, the clinical and prognostic characteristics of the patients have not been systematically assessed. In the current study, we systematically investigated the literature reporting MFS manifestation after receiving the COVID-19 vaccines to provide detailed demographic, clinical, and para-clinical characteristics of these patients to aid healthcare providers in diagnosing MFS sooner and getting better outcomes.

## Material and methods

### Databases search

On January 14, 2023, we performed a thorough search using electronic medical subject headings (MeSH), Embase subject headings (Emtree), and free keywords in PubMed, Web of Science, and Embase to identify relevant studies. The three databases were searched using the following terms without language filter or publication date or type restrictions: "Miller Fisher syndrome", "Fisher syndrome", "Guillain-Barré Syndrome", "ophthalmoplegia", "oculomotor motility disorder", "ataxia", "areflexia", "cranial nerve diseases", "Acute Inflammatory Demyelinating Polyneuropathy", "COVID-19", "SARS-CoV-2", "COVID-19 vaccines", "ChAdOx1 nCoV-19", "2019-nCoV vaccine mRNA-1273", "Moderna vaccine", "BNT162 vaccine", "Pfizer and BioNTech vaccine", "Baiya SARS-CoV-2 VAX COVID-19 vaccine", "Sinovac COVID-19 vaccine", "Sinopharm", "AstraZeneca vaccine", and "Johnson and Johnson vaccine". The exact search strategy and the number of records are provided in a Supplemantary file. Furthermore, Google Scholar was searched manually on the same date to have a sensitive search. The reference lists of the selected articles were also systematically reviewed to further find the relevant articles.

The study was conducted according to PRISMA guidelines [14].

### Final enrollment of studies

The titles and abstracts of the retrieved articles were curated twice by four researchers (D.A., F.A., A.S., and F.S.) independently. In case of disagreement, another author checked the articles. No automation tools were used to exclude or include the records in the process. For the final inclusion and exclusion, researchers independently screened the full texts of the articles that were included in the previous screening step twice. In case of disagreement, another author was consulted.

The following inclusion criteria applied to include the articles: having original data, reporting symptoms of MFS that appeared after a COVID-19 vaccine injection regardless of vaccine type, and providing adequate clinical details of the MFS diagnosis, disease manifestation and information about the patient that we could evaluate the syndrome from the article. Other types of literature (e.g., correspondence, reviews, letters to the editors, expert opinions), studies with original data that reported different subtypes of GBS, and studies with insufficient patient data were considered to be excluded from our study.

Full texts of final studies were thoroughly examined to assess the quality and the risk of bias, and extract the needed data. Three researchers independently extracted the data from the final eligible studies, with discrepancies checked by another researcher (D.A). No automation tools were used to obtain the information.

### Data extraction and risk of bias assessment

We designed a data extraction form to collect the information related to the patients (e.g. age, gender, nationality, history of recent infection, history of COVID-19 infection, and history of non-infectious diseases), the injected vaccine details (e.g. the name of the vaccine and vaccine doses received before the symptom onset), the disease symptoms (e.g. signs and symptoms and abnormal laboratory or imaging findings), and the treatment (treatment regimen, time from symptom onset to treatment, duration of treatment, outcome, and duration from the onset to outcome or length of hospital stay). In the current study, we reported the outcome as two categories; complete recovery, if the researchers reported that the patient's symptoms were completely resolved at the time of the discharge or during follow-up; or improvement, if the researchers reported the patient as partially improved or stated improvement of the sign or symptoms with some remained adverse effects as the last provided information.

To assess the risk of bias and the quality of the included studies, we used the Joanna Briggs Institute (JBI) critical appraisal tools 2020 version for evaluating the included case reports [15] and the case series [16]. Detailed information about the tools and the institute can be sought elsewhere. In short, JBI is an organization providing assessment tools for different types of studies and aids in researching evidence-based medicine [17]. To include studies, case reports in this review had to fulfill at least six criteria out of 8, and the included case series fulfilled 8 out of 10 criteria.

## Results

**Fig. 1:**
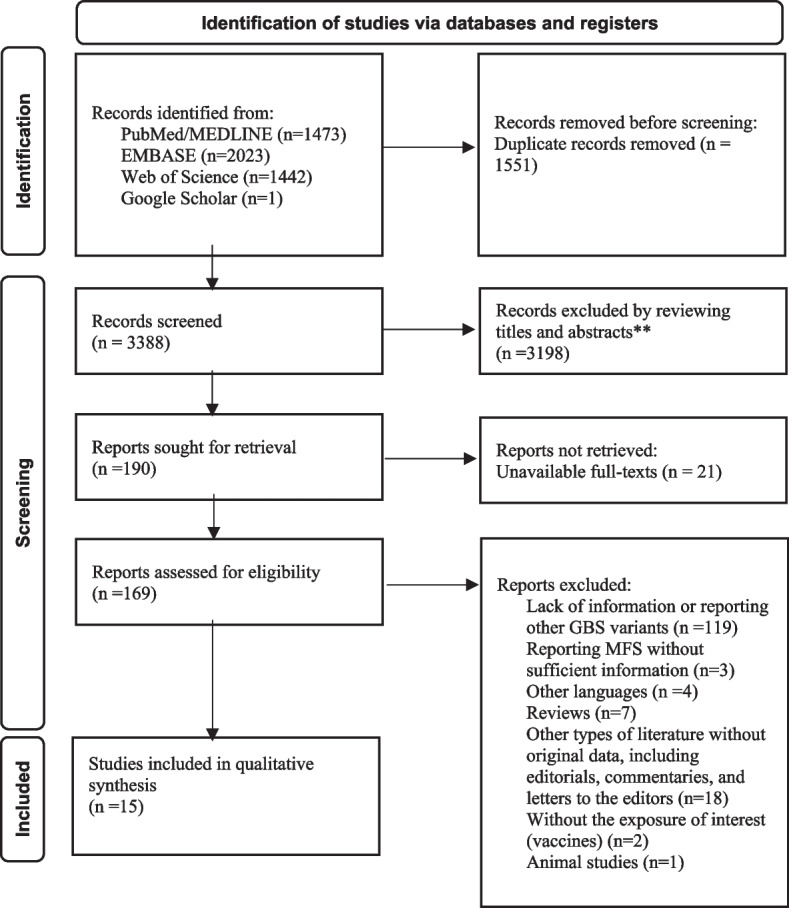
Flow chart of included studies with detailed exclusion reasons (PRISMA Flow Diagram)

A total number of 4939 were retrieved. After removing 1551 duplicated records, titles and abstracts of 3388 articles were screened based on the criteria. After going through titles and abstracts, 190 records were sought for retrieval, and 169 records remained for the full-text assessment. Three studies reported a number of post-vaccination MFS cases. However, they were considered for exclusion due to the lack of information provided for patients. Other reasons for exclusion can be found in Fig. 1.

In this study, after assessing the articles for eligibility, a total number of 15 subjects who developed MFS after receiving COVID-19 vaccines were included from 15 studies, including 14 case reports and one case series, each providing one patient with MFS [18–32]. The median age of the patients was 64 years and ranging between 24 to 84 years. Both the oldest [26] and the youngest [18] patients were females (84 and 24-year-olds); however, most of the patients (66.6%) were men [19–23, 25, 27, 29, 31, 32]. Four cases (26.7%) were reported from Japan [20, 22, 29, 32] and four from the United States [21, 24, 25, 28]. Other cases were from Pakistan [31], South Korea [26], China [24], Australia [19], Croatia [18], Belgium [23], and Brazil [30]. The clinical and demographic characteristics of the patients are presented in Table 1 and the summary of provided data is shown in Table 2.

**Table 1:** Demographic and clinical characteristics of patients with MFS after COVID-19 vaccination (*n* = 15)

Case No	Authors	Country	Study type	Gender/age	Vaccine/dose	Onset time from vaccine to diagnosis (day)	Ocular manifestation	Affected side	Ataxia	Reflex	LP finding
1	Abičić et al. 2021[18]	Croatia	Case report	Female/24	Pfizer/1	18 days	Horizontal diplopia, limited elevation and abduction	Bilateral	-	Normal	Albuminocytological dissociation
2	Dang et al. 2021[19]	Australia	case report	Male/63	AstraZeneca/1	14 days	Diplopia, impaired adduction, restricted upward and downward gaze, incomplete eye closure	Bilateral	+	Absent	Albuminocytological dissociation
3	Kubota et al. 2021 [20]	Japan	case report	Male/65	Pfizer/2	17 days	Ptosis, diplopia, limited adduction, restricted vertical gaze, asymmetric pupils	Right	-	Normal	Albumiocytological dissociation
4	Michaelson et al. 2021 [21]	United States	case report	Male/78	Pfizer/2	14 days	Diplopia, limited eye movement	Bilateral	+	Absent	Albuminocytological dissociation
5	Nishiguchi et al. 2021[22]	Japan	case report	Male/71	Pfizer/1	18 days	Ptosis, ocular pain, absent pupillary reflex	Bilateral	+	Normal	Albuminocytological dissociation
6	Sansen et al. 2021[23]	Belgium	case report	Male/65	Pfizer/1	35 days	Diplopia, abducens nerve palsy, and restricted abduction	Left	+	Absent	Albuminocytological dissociation
7	Chen et al. 2022 [24]	United States	Case report	Female/ in the 70s	Pfizer/2	14 days	Ptosis, diplopia, ophthalmoparesis, restricted saccade movements, reduced pupils response	Bilateral	+	Preserved	Albuminocytological dissociation
8	Kaur et al. 2022 [25]	United States	case report	Male/32	Johnson & Johnson/NR	18 days	Blurry vision, eye pain, inability to fully close the eyes	Bilateral	NR	NR	Albuminocytological dissociation
9	Kim et al. 2022 [26]	South Korea	Case series	Female/84	Astrazeneca/1	8 days	Ptosis, diplopia	Bilateral	+	Absent	Albuminocytological dissociation
10	Liang et al. 2022 [27]	China	Case report	Male/64	Sinovac-CoronaVac/2	12 days	Diplopia, limited abduction, inability to fully close the right eye	Bilateral, more prominently, right	+	Absent	Albuminocytological dissociation
11	Loza et al. 2021[28]	United States	Case report	Female/60	Johnson & Johnson/1	10 days	Diplopia, limited abduction	Bilateral	-	Absent	Albuminocytological dissociation
12	Nanatsue et al. 2022 [29]	Japan	Case report	Male/72	Moderna/2	7 days	Diplopia and limited binocular abduction	Bilateral	+	Absent	NR
13	Pirola et al. 2022 [30]	Brazil	Case report	Female/47	AstraZeneca/1	7 days	Absent ophthalmoplegia	-	+	NR	Albuminocytological dissociation
14	Siddiqi et al. 2022 [31]	Pakistan	Case report	Male/53	Sinovac-CoronaVac/1	8 days	Abducens nerve palsy, inability to fully close the eyes	Right	+	Absent	Albuminocytological dissociation
15	Yamakawa et al. 2022 [32]	Japan	Case report	Male/30	Pfizer/2	7 days	Diplopia, lateral gaze palsy	Bilateral	+	Absent	Normal

Case No	Antibodies	Imaging	NCS and EMG	Signs, symptoms, or examination findings	Past History of covid infection/ recent infection/non-infectious diseases	Time from symptom onset to treatment	Duration of treatment/hospital stay	Duration to outcome	Treatment	Outcome
1	+ anti-GQ1b + anti-PCNA Speckled ANA	Normal	Normal EMG	NR	Negative/ Negative /Negative	NR	3, and 5 days/NR	3 weeks (after treatment)	IV methylprednisolone (500 mg daily, with no improvement), IVIG (2 g/kg)	Improved
2	- anti-GQ1b	Bilateral facial and oculomotor nerve enhancement in MRI	Axonal neuropathy with reduced amplitude	Lower back pain radiating to the legs, bilateral facial weakness, facial diplegia, lower and upper limb weakness, walking difficulty, paresthesia	NR/NR/ Negative	Three days	5 days IVIg, 4 weeks rehabilitation/NR	6 weeks (after onset)	IVIg (2g/kg) and topical lubricating eye drop, inpatient rehabilitation	Improved
3	+ anti-GQ1b	Enhanced right oculomotor nerve	Normal	-	Negative/NR/DM, glaucoma, BPPV	31 days	5/18 days	54 days (after onset)	IVIg (0.4 g/kg)	Complete resolution
4	Equivocal anti-GQ1b	Normal	NR	Paresthesia and dysmetria	NR/positive/chronic painless diplopia, Inflammatory pseudotumor	2 weeks	5 days/NR	3 days (after treatment)	IVIg (2g/kg)	Improved
5	- anti-GQ1b	Minor venous dilation of the middle cranial fossa in MRI, Normal MR angiography	Normal	Headache	Negative/Negative/ DM recovered diabetic ophthalmoplegia	29 days	2 × 5 day IVIg/ 51 days	88 days (after onset)	Two courses of IVIg	Complete resolution
6	- anti-GQ1b	Normal	NR	Left deviation of the body	Negative/Negative/DM and prostatic hyperplasia	2 days	5 days/NR	2 weeks (after treatment)	IVIg (0.4 g/kg)	Improved
7	+ anti-GQ1b	NR	Acute left facial neuropathy, reduced fibular and tibial motor amplitudes, and a mildly prolonged ulnar F wave relative to the F estimate	Progressive gait imbalance, blood pressure fluctuations	Negative/Negative / HTN, remitting breast cancer	NR	5 days/NR	Two months (after discharge)	IVIg (0.4 g/kg)	Improved
8	- anti-GQ1b	Normal MRI	NR	Dysarthria, facial diplegia, and inability to raise eyebrows and smile	NR/NR/-	NR	NR	NR	IVIg, ICU admitted	Improved
9	+ anti-GQ1b	NR	Normal	NR	None/none/HTN	NR	NR	30 (after onset)	No treatment	Improved
10	+ anti-GQ1b + anti-GT1b	Normal	axonal neuropathy, Negative single-muscle-fiber electromyogram	Paresthesia, dizziness, nausea, headache, right facial palsy, dribbling of saliva	NR/negative/cholecystitis and chronic gastritis	21 days	2 days IVIg,20 days acupuncture/7 days	54 days (after onset)	IVIg (0.4 g/kg), acupuncture (once a day)	Complete resolution
11	- anti-GQ1b	Cauda equine enhancement	Absent F wave and H wave, neurogenic recruitment	Nausea, vomiting, headache, back pain, leg pain, bilateral facial weakness	NR/negative/migraines	4 days	2/10 days	10 days (after admission)	IVIg (2gr/kg)	Improved
12	+ anti-GQ1b	Normal	F wave abnormalities	Right-sided peripheral facial nerve palsy	Negative/Negative/HTN, hyperuricemia, and hyperlipidemia	2 days	NR/22 days	1 month (after discharge)	IVIg, steroid, valacyclovir, and mecobalamin	Complete resolution
13	NR	Normal head CT at the admission and mineral deposition in the basal ganglia one week prior to the admission	motor and sensory demyelinating polyneuropathy, acute polyradiculoneuritis	Dysphonia, lower and upper limbs weakness, peripheral facial diparesis	Negative/flu/NR	NR	5 days /NR	At the discharge	IVIg (0.4 g/kg), gabapentin 300 mg BID and motor physiotherapy after discharge	Improved
14	Not performed	Normal	Prolonged latency with reduced conduction velocities F wave abnormalities	Paresthesia, lower limbs myalgia, and reduced proprioception, positive Romberg sign, right-sided lower motor neuron facial palsy, and dribbling of saliva	Negative/Negative/HTN, IHD, smoker	2 days	6 weeks outpatient physiotherapy/NR	10 weeks (after discharge)	Pregabalin 50mg and physiotherapy	Complete resolution
15	+ anti-GQ1b + anti-GT1a	Normal	Normal	Dizziness, walking difficulty	NR/NR/NR	5 days	5 days /NR	93 days (from treatment)	IVIg (0.4 g/kg/day) for 5 days	Complete resolution

*NCS* nerve conduction study, *EMG* electromyography, *CT* computed tomography, *MRI* Magnetic resonance imaging, *NR* Not reported, *LP* lumbar puncture, *HTN* hypertension, *DM* diabetes mellitus, *IHD* ischemic heart disease, *IVIg* intravenous immunoglobulin, *BPPV* benign paroxysmal positional vertigo

**Table 2:** Summary of clinical characteristics and outcomes

**Characteristics**		**Total cases**
**Age, years, median [min–max]**	64 [24–84]	15
**Sex (%)**		15
Female	5 (33.3%)	
Male	10 (66.7%)	
**Comorbidities**		12
Hypertension	4 (33.3%)	
Diabetes mellitus	3 (25.0%)	
IHD	1 (8.3%)	
Glaucoma	1 (8.3%)	
**Region (%)**		15
East Asia	6 (40.0%)	
United States	4 (26.7%)	
Europe	2 (13.3%)	
MENA region	1 (6.7%)	
Australia	1 (6.7%)	
South America	1 (6.7%)	
**Time from vaccination to symptom onset (%)**		15
0–10 days	6 (40.0%)	
11–20 days	8 (53.3%)	
21 days or more	1 (6.7%)	
**Vaccine name (%)**		15
Pfizer and BioNTech	7 (46.7%)	
Astra Zeneca	3 (20.0%)	
Johnson & Johnson	2 (13.3%)	
Sinovac	2 (13.3%)	
Moderna	1 (6.7%)	
**Vaccine dose (%)**		14
First	8 (57.1%)	
Second	6 (42.9%)	
**Clinical findings (%)**		15
Ataxia	11 (73.3%)	
Areflexia	9 (60.0%)	
Facial nerve palsy/facial weakness	7 (46.7%)	
Headache	3 (20.0%)	
Limbs weakness	2 (13.3%)	
Dizziness	2 (13.3%)	
Dysarthria	1 (6.7%)	
**Ocular manifestations (%)**		
Diplopia	11 (73.3%)	15
Ptosis	4 (26.7%)	
Restricted abduction	6 (40.0%)	
Inability to fully close one or two eyes	4 (26.7.0%)	
Absent or abnormal pupil reflex	3 (20.0%)	
**Side of ocular manifestations (%)**		
Bilateral	11 (78.6%)	14
Right	2 (14.3%)	
Left	1 (7.1%)	
**Antibodies profile**		13
Positive anti-GQb1	7 (53.8%)	
Equivalent anti-GQb1	1 (7.7%)	
**NCS and EMG**		12
Abnormal findings	7 (58.3%)	
Normal	5 (41.7%)	
**Treatment (%)**		15
IVIg	13 (86.6%)	
Physiotherapy	2 (13.3%)	
Steroid	1 (6.7%)	
**Outcome (%)**		15
Complete resolution	6 (40.0%)	
Improved	9 (60.0%)	

*MENA* Middle East and North Africa, *NCS* Nerve Conduction Study, *EMG* Electromyography

Most case reports fulfilled all 8 criteria, and most case series got 8 out of 10 questions. The details for each case report are provided in Table 3 and the case series are presented in Table 4.

**Table 3:** Quality assessment of the case reports using the JBI Critical Appraisal Checklist

Authors	1. Were patient’s demographic characteristics clearly described?	2. Was the patient’s history clearly described and presented as a timeline?	3. Was the current clinical condition of the patient on presentation clearly described?	4. Were diagnostic tests or assessment methods and the results clearly described?	5. Was the intervention(s) or treatment procedure(s) clearly described?	6. Was the post-intervention clinical condition clearly described?	7. Were adverse events (harms) or unanticipated events identified and described?	8. Does the case report provide takeaway lessons?	Total yes
**Abičić et al. 2022** [18]	Yes	Yes	Yes	Yes	Yes	Yes	Yes	Yes	8
**Dang et al. 2021** [19]	Yes	Yes	Yes	Yes	Yes	Yes	Yes	Yes	8
**Kubota et al. 2021** [20]	Yes	Yes	Yes	Yes	Yes	Yes	Yes	Yes	8
**Michaelson et al. 2021** [21]	Yes	Yes	Yes	Yes	Yes	Yes	Yes	Yes	8
**Nishiguchi et al. 2021** [22]	Yes	Yes	Yes	Yes	Yes	Yes	Yes	Yes	8
**Sansen et al. 2021** [23]	Yes	Yes	Yes	Yes	Yes	Yes	Yes	Yes	8
**Chen B, et al. 2022 **[24]	Yes	Yes	Yes	Yes	Yes	Yes	Yes	Yes	8
**Kaur et al. 2022** [25]	Yes	Yes	Yes	Yes	Yes	No	Yes	Yes	7
**Liang et al. 2022** [27]	Yes	Yes	Yes	Yes	Yes	No	No	Yes	6
**Loza et al. 2021** [28]	Yes	Yes	Yes	Yes	Yes	Yes	Yes	Yes	8
**Nanatsue et al. 2022** [29]	Yes	Yes	Yes	Yes	Yes	Yes	Yes	Yes	8
**Pirola et al. 2022** [30]	Yes	Yes	Yes	Yes	Yes	Yes	Yes	Yes	8
**Siddiqi et al. 2022** [31]	Yes	Yes	Yes	Yes	Yes	Yes	Yes	Yes	8
**Yamakawa et al. 2022** [32]	Yes	No	Yes	Yes	Yes	Yes	Yes	Yes	7

**Table 4:** Quality assessment of the case series using the JBI Critical Appraisal Checklist

Title	1. Were there clear criteria for inclusion in the case series?	2. Was the condition measured in a standard, reliable way for all participants included in the case series?	3. Were valid methods used for identification of the condition for all participants included in the case series?	4. Did the case series have consecutive inclusion of participants?	5. Did the case series have complete inclusion of participants?	6. Was there clear reporting of the demographics of the participants in the study?	7. Was there clear reporting of clinical information of the participants?	8. Were the outcomes or follow up results of cases clearly reported?	9. Was there clear reporting of the presenting site(s)/clinic(s) demographic information?	10. Was statistical analysis appropriate?	Total yes
**Kim et al. 2022** [26]	Yes	Yes	Yes	Yes	Yes	Yes	No	Yes	Yes	Not applicable	8

### Narrative synthesis

Pfizer made up 46.7% (7/15) of the injected vaccines [18, 20–24, 32]. The next most frequent vaccine received was Astra Zeneca, with 20.0% (3/15) patients [19, 26, 30]. Each of Sinovac [27, 31] and Johnson & Johnson injection [25, 28] were reported in two patients (13.3%), and in only one study with one patient Moderna was received [29]. Among the documented cases, eight patients had reported the syndrome onset after the first dose of vaccination (53.3%) [18, 19, 22, 23, 26, 28, 30, 31], and six patients with a second dose (40.0%) [20, 21, 24, 27, 29, 32]. The information was not provided in one case [25].

The median latency period from vaccination to symptoms onset was 14 days ranging from 7 to 35 days. More than half of the included studies (8/15, 53.3%) reported a duration between 11 and 20 days following the immunization to the onset of symptoms [18–22, 24, 25, 27]. While six of them (40.0%) reported less duration from vaccination to syndrome manifestation [26, 28–32], and only one study claimed it took 35 days [23].

Concerning the MFS triad assessment, only one study did not report any ocular complaint [30]. When evaluating the prevalence of different ocular manifestations of the syndrome, diplopia was the most frequent symptom, which affected 11 individuals (73.3%) [18–21, 23, 24, 26–29, 32]. Six studies (40.0%) reported restricted abduction or abducens nerve palsy [18, 23, 27–29, 31]. Ptosis was presented in four individuals (25%) [20, 22, 24, 26]. Three patients had abnormal pupil reflexes [20, 22, 24], and two patients reported being unable to completely close one or both eyes [27, 31]. Of these ocular signs, 78.3% (11/14) were bilateral [18, 19, 21, 22, 24–29, 32]. Moreover, 11 patients (73.3%) had ataxia [19, 21–24, 26, 27, 29–32], and reflexes were absent in nine (60.0%) [19, 21, 23, 26–29, 31, 32]. Eight patients (53.3%) had the complete triad of MFS [19, 21, 23, 26, 27, 29, 31, 32]. One or two features were absent in the rest.

Among other neurological conditions, facial weakness or facial nerve palsy was the most frequent complication that was reported in seven subjects [19, 25, 27–31]. Four patients had paresthesia [19, 21, 27, 31], and three had headaches [22, 27, 28]. Limb weakness [19, 30], nausea [24, 28], back pain [19, 28], and dizziness [27, 32] were each reported in two patients. One patient had dysarthria [25], and blood pressure fluctuation was diagnosed in one [24].

In terms of comorbidities and other medical conditions, four patients mentioned a medical history of hypertension (HTN) [24, 26, 29, 31], and three listed diabetes mellitus (DM) [20, 22, 23]. Importantly, one patient was admitted with flu [30], and one had positive testing for COVID-19 infection [21]. Other conditions were glaucoma and benign paroxysmal positional vertigo (BPPV) in one patient [20] and hyperuricemia and hyperlipidemia in another subject [29].

For paraclinical examinations, lumbar puncture (LP) was reported to be performed in all but one patient [30]. Albuminocytological dissociation was detected in 13 individuals [18–28, 30, 31], and one patient's LP analysis was normal [32]. Anti-Ganglioside Q1b (Anti-GQ1b) was positive in seven patients [18, 20, 24, 26, 27, 29, 32] and equivalent in one person [21]. The anti-GT1a [32], anti-GT1b [27], and anti-PCNA [18] were each positive in one patient.

Among the studies that reported the results of electromyography (EMG) or nerve conduction studies (NCS), abnormalities were reported in more than half (7/12, 58.3%) of studies [19, 24, 27–31]. In four cases (33.3%), abnormal F waves were observed [24, 28, 29, 31], that all were within the first ten days; at the time of admission [24], two [28], three [29], and ten days [31] after admission.

Thirteen patients underwent imaging, of which only four cases (30.7%) had modest abnormalities, including slight venous dilatation of the middle cerebral fossa in magnetic resonance imaging (MRI) [22], enhanced right oculomotor nerve [20], bilateral facial and oculomotor nerve in MRI [19], and Cauda equina enhancement [28].

Regarding the treatment, intravenous immunoglobulin (IVIg) was the most frequently used treatment (13/15) [18–25, 27–30, 32]. Six patients received 0.4 g/kg [20, 23, 24, 27, 30, 32], of five of them for five days (2g/kg as total dose) [20, 23, 24, 30, 32], and one patient for two days [27]. The four others had 2 g/kg [18, 19, 21, 28], three of them for five days (10g/kg as total dose) [18, 19, 21, 28], and one patient for two days [28]. Three studies did not report the dosage [22, 25, 29]. Besides IVIg, two patients also received steroids [18, 29] which were reported not to be effective in one patient [18]. Two patients had physiotherapy [30, 31], one received valacyclovir and mecobalamin [29], and one had daily acupuncture sessions [27]. One patient received pregabalin and physical therapy [29], whereas the other received no care [26]. Six patients completely benefited from the treatment, while the nine remaining only showed signs of improvement at the last session.

Hospital stays ranged in duration from 5 to 51 days, and the outcomes were assessed at the time of the discharge or during the follow-up, from the shortest three to 93 days after receiving the treatment, as the longest duration.

In terms of the prognosis, signs and symptoms of 40.0% (6/15) of patients were reported to be completely resolved at the last time of the follow-up. The rest (9/15, 60.0%) of patients improved and some of adverse effects were reported to be still present as the last record of the case. Although no fatal outcome was reported, it took some weeks [21, 27] and even one month [20, 22] for some patients to be diagnosed with MFS cases and get proper treatment.

## Discussion

In this systematic review, we characterized 15 cases that developed MFS after receiving COVID-19 vaccination. The median age of patients was 64; most were male. East Asia made up the area with the most report. In most cases, the latency between receiving vaccines and symptoms onset was 11–20 days. Diplopia was the most frequent complaint, and ocular manifestations were bilateral rather than one-sided. IVIg was the most administered treatment regimen, with 0.4g/kg for five days. No fatal outcome was reported, and all subjects experienced improvement, with 40% being reported as having completely resolved signs and symptoms at the discharge or the last follow-up.

Importantly, in terms of the epidemiology, our results were consistent with the findings of the previous systematic review of post-COVID-19 infection MFS cases that most patients were middle-aged males [33]. Furthermore, in our study, the male:female ratio was 2:1, which is also in line with the previous works on MFS [2, 34].

In this review, Pfizer/BioNTech, an mRNA vaccine, was the most frequently reported vaccine, with seven out of 15 cases. Importantly, the rates of MFS after Pfizer, Johnson & Johnson, and Moderna in the United States are previously shown to be in the expected incidence range; therefore, there is a possibility that the type of vaccine does not make a significant difference [13]. However, it is noteworthy that vaccines of messenger RNA export spike proteins on the cell that provoke the production of antibodies and T-cell reactions [35]. Even though the current evidence is limited, it is suggested that these immunological changes may cause producing virus neutralizing as well as anti-GQ1b antibodies, therefore leading to neurological complications [20]. Importantly, the main suggested mechanisms by which COVID-19 vaccine triggers autoimmunity include molecular mimicry, the production of particular autoantibodies and the existence of certain vaccine adjuvants. The similarity between specific vaccine components and certain human proteins could trigger immune cross-reactivity and lead the immune system against pathogenic antigens to attack similar proteins in susceptible population [36]. In general, after mRNA vaccine injection, the ability to simultaneously activate the humoral and cellular immune systems may explain the side effects [37].

Among neurological signs and symptoms, other than the MFS triad, facial nerve palsy was the most common side effect, followed by dizziness, headache, myalgia, and paresthesia. Similar to our study, these signs and symptoms were reported as the most common neurologic side effects of the COVID-19 vaccines [12, 38]. GBS is also among the most common side effects of vaccines [39], which must be paid attention to when listing differential diagnoses in patients with neurological complaints and the history of a recent COVID-19 vaccination. Although GBS provides no absolute contraindication to receive COVID‐19 vaccines, it is suggested as an adverse event attributed to the vaccine, of which the likelihood of a causal link with vaccination should be determined through assessment along with getting detailed information about the complication and the severity of the condition [40].

MFS patients who present with limb weakness are considered MFS-GBS overlap syndromes [41–43]. Although no early predictors for progression from MFS to MFS-GBS overlap syndrome are provided, it is suggested that the transition occurs in the first week of the syndrome onset which warrants the need to carefully monitor MFS cases for at least the first week [43]. In our study, among 15 patients, two subjects reported limb weakness [19, 30]. Both improved and benefited from IVIg. Although the interval from symptom onset to the treatment of one patient was not provided [30], the other received treatment three days after the onset of the symptom [19]. Therefore, based on the current data of included studies and in line with previous reports, we support the benefit of early diagnosis and treatment of MFS to avoid further complications and progressive symptoms.

The cerebrospinal fluid (CSF) analysis is reported to be done in the MFS diagnosis, in which an increase in albuminocytological dissociation can be seen in 90% of patients [34]. However, this finding can not differentiate MFS from GBS [44]. In this study, and in line with previous studies [33, 34], 92.3% of patients showed albuminocytological dissociation in their CSF analysis, and only one study reported normal findings.

Treatment of MFS is generally supportive, using pain relievers and, if necessary, respiratory support. Alhough no randomized clinical trials have been performed for the MFS treatment, corticosteroids either orally or by injection have been reported not to be effective [33]. Similarly, in one patient of our study, before IVIg receiving, daily 500 mg of methylprednisolone showed no improvement, and subsequently, the patient went on IVIg therapy [18].

IVIg and plasmapheresis are effective treatments for severe MFS. No difference in the treatment outcomes is reported. However, the former is preferred for its convenience and fewer adverse effects [33]. In our review study, IVIg was administered to 86.6% of cases. All these patients experienced improvement mostly on the second day of the therapy. One patient did not receive treatment and exhibited improvement and a favorable outcome 30 days after the onset of the symptoms [26], and one had completely resolved symptoms only with pregabalin and physiotherapy [31].

Although among 15 patients, no death was reported, and all patients improved either partially or completely, the long duration from the onset to the treatment of some patients shows a challenging diagnosis. According to the current literature, the progressive nature of the syndrome and especially if the patient does not present with the classic triad, might make the diagnosis difficult and cause delay in receiving the proper treatment [34].

As mentioned earlier, simultaneous activation of the humoral and cellular immune systems following the injection of mRNA vaccines may cause MFS after vaccination [20, 35]. Another possible mechanism is the antibody cross-reaction [39]. Considering that COVID-19 vaccines induce immunization against the virus spike proteins, proteins might bind to sialic acid-containing glycoprotein and gangliosides on cell surfaces, which might explain a potential association [45]. Although MFS is reported that can be caused by COVID-19 infection [33], there is no solid evidence linking COVID-19 vaccination as a result of MFS exposure and demonstration. Furthermore, using case-centered and self-controlled case series analysis, Lee and colleagues rejected the proposed definitive and causal relationship between COVID-19 vaccination and the development of MFS [13]. One included study in this systematic review was a report of a COVID-19 vaccine clinical trial in which the side effects have also been observed in one subject of the placebo group [28]. Due to the design of the included studies, which are case reports and case series, we can not approve nor reject a potential casualty. However, regardless of the type of association, it is worth studying the characteristics of patients to provide better insight for clinicians and improve prognosis, as we did in this systematic review.

One of the limitations of our study is that several studies on patients with GBS did not address the subtype of the disease nor provide enough clinical features of GBS in each patient; therefore, we suggest that data on MFS cases are unavailable due to the lack of provided patients clinical characteristics. Furthermore, the currently available data on MFS cases after COVID-19 vaccination are reports of cases with no control group, and since the case–control studies are needed to get further information, In this review, we could not provide the incidence rate of the disease and compare it before and after COVID-19 vaccination.

To our knowledge, this review is the first systematic review studying demographic characteristics of patients with MFS after COVID-19 vaccination. Furthermore, we tried to provide details and clues based on current evidence to aid healthcare providers in the early diagnosis and treatment of patients with neurological manifestations and subsequently reduce its consequences and improve the quality of their lives.

## Conclusion

According to our findings, MFS that occurred following SARS-Cov2 immunization is a neurological condition with a good prognosis and favorable outcomes. Male patients, especially in their 60s and 70s, who came with a history of previous COVID-19 vaccination and complaints of ophthalmoplegia, ataxia, and areflexia, should be better suspected for MFS. The MFS and its approach must be among the diseases at the top of the differential diagnosis list of a physician in order not to miss the opportunity to provide the patient with well-established treatment regimens, and to avoid the consequences of this disease progression. The exact cause of its occurrence has not been determined well, and underlying pathologies need to be further evaluated. However, based on up-to-date findings of other investigations it can be concluded that molecular mimicry and vaccine components and certain human proteins similarity might trigger this syndrome in susceptible population and the symptoms diminish following the reduction in anti-GQ1b antibody. Despite the above, vaccination should still be advocated because its risk–benefit ratio remains positive when compared to COVID-19 serious adverse events.

### Supplementary Information

**Additional file 1.**

## Data Availability

Data could be available from the corresponding author upon reasonable reply.

## Abbreviations

MFS: Miller-Fisher syndrome
GBS: Guillain–Barré syndrome
DPT: Diphtheria—Pertussis—Tetanus
JBI: Joanna Briggs Institute
HTN: Hypertension
DM: Diabetes mellitus
LP: Lumbar puncture
EMG: Electromyography
NCS: Nerve conduction studies
CT: Computed tomography
MRI: Magnetic resonance imaging
IVIg: Intravenous immunoglobulin
BPPV: Benign paroxysmal positional vertigo
IHD: Ischemic heart disease
MENA: Middle East and North Africa
