# Supplementary material for: Clinical characteristics and prognosis of temporary miller fisher syndrome following COVID-19 vaccination: a systematic review of case studies
Source: BMC Neurol. 2023 Sep 21;23:332. doi: 10.1186/s12883-023-03375-4 (PMC10512542; doi:10.1186/s12883-023-03375-4)
Supplement: Supplementary file 1 — Additional file 1. [file 12883_2023_3375_MOESM1_ESM.docx]

**PubMed**

| Search | Query | Results |
| --- | --- | --- |
| #1 | ((((((((((((((((Miller Fisher Syndrome[MeSH Terms]) OR (Miller Fisher Syndrome[Title/Abstract])) OR (Miller Fisher*[Title/Abstract])) OR (Miller-Fisher Syndrome[Title/Abstract])) OR (Miller-Fisher*[Title/Abstract])) OR (Fisher syndrome[Title/Abstract])) OR (Guillain-Barre Syndrome[MeSH Terms])) OR (Guillain-Barre Syndrome[Title/Abstract])) OR (ophthalmoplegia[Title/Abstract])) OR (ataxia[Title/Abstract])) OR (areflexia[Title/Abstract])) OR (oculomotor motility disorder[Title/Abstract])) OR (Ocular Motility Disorders[MeSH Terms])) OR (cranial nerve diseases[MeSH Terms])) OR (cranial nerve diseases[Title/Abstract])) OR (Acute Inflammatory Demyelinating Polyneuropathy[Title/Abstract])) OR (Guillain Barre[Title/Abstract]) | 175,565 |
| #2 | (((((((((((((((((((((((((((((((COVID-19[MeSH Terms]) OR (COVID-19[Title/Abstract])) OR (SARS-CoV-2[MeSH Terms])) OR (SARS-CoV-2[Title/Abstract])) OR (COVID-19 Vaccines[MeSH Terms])) OR (COVID-19 Vaccin*[Title/Abstract])) OR (ChAdOx1 nCoV-19[MeSH Terms])) OR (ChAdOx1 nCoV-19[Title/Abstract])) OR (2019-nCoV Vaccine mRNA-1273[MeSH Terms])) OR (2019-nCoV Vaccine mRNA-1273[Title/Abstract])) OR (BNT162 Vaccine[MeSH Terms])) OR (BNT162 Vaccine[Title/Abstract])) OR (Baiya SARS-CoV-2 VAX COVID-19 vaccine[MeSH Terms])) OR (Baiya SARS-CoV-2 VAX COVID-19 vaccine[Title/Abstract])) OR (sinovac COVID-19 vaccine[MeSH Terms])) OR (sinovac COVID-19 vaccine[Title/Abstract])) OR (Ad26COVS1[MeSH Terms])) OR (Ad26COVS1[Title/Abstract])) OR (AstraZeneca vaccine[MeSH Terms])) OR (AstraZeneca vaccine[Title/Abstract])) OR (Moderna vaccine[MeSH Terms])) OR (Moderna vaccine[Title/Abstract])) OR (Pfizer BioNTech vaccine[Title/Abstract])) OR (pfizer-biontech vaccine[Title/Abstract])) OR (Johnson & Johnson vaccince[Title/Abstract])) OR (COVID-19 vaccine booster shot[MeSH Terms])) OR (COVID-19 vaccine booster shot[Title/Abstract])) OR (Bharat Biotech[Title/Abstract])) OR (Sinopharm[Title/Abstract])) OR (Covaxin[Title/Abstract])) OR (Novavax[Title/Abstract])) OR (CovoVax[Title/Abstract]) | 322,284 |
| #1 AND #2 | (((((((((((((((((Miller Fisher Syndrome[MeSH Terms]) OR (Miller Fisher Syndrome[Title/Abstract])) OR (Miller Fisher*[Title/Abstract])) OR (Miller-Fisher Syndrome[Title/Abstract])) OR (Miller-Fisher*[Title/Abstract])) OR (Fisher syndrome[Title/Abstract])) OR (Guillain-Barre Syndrome[MeSH Terms])) OR (Guillain-Barre Syndrome[Title/Abstract])) OR (ophthalmoplegia[Title/Abstract])) OR (ataxia[Title/Abstract])) OR (areflexia[Title/Abstract])) OR (oculomotor motility disorder[Title/Abstract])) OR (Ocular Motility Disorders[MeSH Terms])) OR (cranial nerve diseases[MeSH Terms])) OR (cranial nerve diseases[Title/Abstract])) OR (Acute Inflammatory Demyelinating Polyneuropathy[Title/Abstract])) OR (Guillain Barre[Title/Abstract])) AND ((((((((((((((((((((((((((((((((COVID-19[MeSH Terms]) OR (COVID-19[Title/Abstract])) OR (SARS-CoV-2[MeSH Terms])) OR (SARS-CoV-2[Title/Abstract])) OR (COVID-19 Vaccines[MeSH Terms])) OR (COVID-19 Vaccin*[Title/Abstract])) OR (ChAdOx1 nCoV-19[MeSH Terms])) OR (ChAdOx1 nCoV-19[Title/Abstract])) OR (2019-nCoV Vaccine mRNA-1273[MeSH Terms])) OR (2019-nCoV Vaccine mRNA-1273[Title/Abstract])) OR (BNT162 Vaccine[MeSH Terms])) OR (BNT162 Vaccine[Title/Abstract])) OR (Baiya SARS-CoV-2 VAX COVID-19 vaccine[MeSH Terms])) OR (Baiya SARS-CoV-2 VAX COVID-19 vaccine[Title/Abstract])) OR (sinovac COVID-19 vaccine[MeSH Terms])) OR (sinovac COVID-19 vaccine[Title/Abstract])) OR (Ad26COVS1[MeSH Terms])) OR (Ad26COVS1[Title/Abstract])) OR (AstraZeneca vaccine[MeSH Terms])) OR (AstraZeneca vaccine[Title/Abstract])) OR (Moderna vaccine[MeSH Terms])) OR (Moderna vaccine[Title/Abstract])) OR (Pfizer BioNTech vaccine[Title/Abstract])) OR (pfizer-biontech vaccine[Title/Abstract])) OR (Johnson & Johnson vaccince[Title/Abstract])) OR (COVID-19 vaccine booster shot[MeSH Terms])) OR (COVID-19 vaccine booster shot[Title/Abstract])) OR (Bharat Biotech[Title/Abstract])) OR (Sinopharm[Title/Abstract])) OR (Covaxin[Title/Abstract])) OR (Novavax[Title/Abstract])) OR (CovoVax[Title/Abstract])) | [1,](https://pubmed.ncbi.nlm.nih.gov/?term=longquery7569eeb53e48689eae9e&size=200&ac=no&sort=relevance)473 |

**Embase**

| Search | Query | Results |
| --- | --- | --- |
| #1 | 'guillain barre syndrome'/exp OR 'guillain barre syndrome' OR 'guillain barre syndrome':ti,ab,kw OR 'miller fisher syndrome':ti,ab,kw OR 'miller-fisher syndrome':ti,ab,kw OR 'miller fisher':ti,ab,kw OR 'fisher syndrome':ti,ab,kw OR 'miller fisher*':ti,ab,kw OR ophthalmoplegia:ti,ab,kw OR ataxia:ti,ab,kw OR areflexia:ti,ab,kw OR 'oculomotor motility disorder':ti,ab,kw OR 'ocular motility disorders':ti,ab,kw OR 'cranial nerve diseases':ti,ab,kw OR 'Demyelinating Polyneuropathy':ti,ab,kw OR 'Guillain Barre':ti,ab,kw | 94,297 |
| #2 | 'covid 19'/exp OR 'covid 19':ti,ab,kw OR 'sars cov 2'/exp OR 'sars cov 2':ti,ab,kw OR 'covid-19 vaccines'/exp OR 'covid-19 vaccin*':ti,ab,kw OR 'vaxzevria'/exp OR vaxzevria:ti,ab,kw OR 'chadox1 ncov-19'/exp OR 'elasomeran'/exp OR elasomeran:ti,ab,kw OR '2019-ncov vaccine mrna-1273':ti,ab,kw OR 'bnt 162 vaccine'/exp OR 'bnt 162 vaccine':ti,ab,kw OR 'baiya sars-cov-2 vax covid-19 vaccine' OR 'coronavac'/exp OR coronavac:ti,ab,kw OR 'sinovac covid-19 vaccine':ti,ab,kw OR 'ad26.cov2.s vaccine'/exp OR ad26covs1:ti,ab,kw OR 'astrazeneca vaccine':ti,ab,kw OR 'moderna vaccine':ti,ab,kw OR 'pfizer biontech vaccine':ti,ab,kw OR 'johnson & johnson vaccince':ti,ab,kw OR 'covid-19 vaccine booster shot':ti,ab,kw OR 'sars-cov-2 vaccine'/exp OR 'sars-cov-2 vaccin*':ti,ab,kw OR 'biotech':ti,ab,kw OR 'sinopharm':ti,ab,kw OR 'covaxin':ti,ab,kw OR 'nvx-cov2373 vaccine':ti,ab,kw OR 'covovax':ti,ab,kw | 380,087 |
| #3 | #1 AND #2 | 2,023 |

Web of science

| Search | Query | Results |
| --- | --- | --- |
| #1 | (((((((((((((ALL=(ophthalmoplegia)) OR ALL=(ataxia)) OR ALL=(areflexia)) OR WC=(Ocular Motility Disorders)) OR WC=(cranial nerve diseases)) OR ALL=(oculomotor dysfunction)) OR ALL=(Miller Fisher)) OR ALL=(Miller-Fisher)) OR ALL=(Guillain Barre)) OR ALL=(Guillain-Barre)) OR ALL=(Fisher Syndrome)) OR ALL=(Ophthalmoplegia, Ataxia and Areflexia Syndrome)) OR ALL=(Acute Autoimmune Neuropathy)) OR ALL=(Acute Inflammatory Demyelinating Polyneuropathy) | 77,024 |
| #2 | (((((((((((((((((ALL=(SARS-CoV-2)) OR ALL=(COVID-19)) OR ALL=(COVID-19 Vaccin*)) OR WC=(SARS-CoV-2)) OR WC=(COVID-19)) OR ALL=(COVID-19 Vaccines)) OR ALL=(SARS Coronavirus 2 Vaccines)) OR ALL=(COVID-19 Vaccine)) OR ALL=(2019-nCoV Vaccines)) OR ALL=(2019 Novel Coronavirus Vaccine)) OR ALL=(2019 Novel Coronavirus Vaccines)) OR ALL=(2019-nCoV Vaccine)) OR ALL=(COVID 19 Vaccine)) OR ALL=(Coronavirus Disease-19 Vaccine)) OR ALL=(Coronavirus Disease-19 Vaccines)) OR ALL=(Coronavirus Disease 2019 Virus Vaccines)) OR ALL=(SARS2 Vaccines)) OR ALL=(COVID19 Virus Vaccines) | 374,423 |
| #3 | #1 AND #2 | 1,442 |
